# Is double-blinding possible while administering fluids in the intensive care unit?

**Published:** 2013-06

**Authors:** Ahmet Baris Durukan, Hasan Alper Gurbuz, Cem Yorgancioglu, Murat Tavlasoglu

**Affiliations:** Department of Cardiovascular Surgery, Medicana International Ankara Hospital, Ankara, Turkey; Department of Biology, Hacettepe University Faculty of Science, Ankara, Turkey; Department of Cardiovascular Surgery, Medicana International Ankara Hospital, Ankara, Turkey; Department of Biology, Hacettepe University Faculty of Science, Ankara, Turkey; Department of Cardiovascular Surgery, Medicana International Ankara Hospital, Ankara, Turkey; Diyarbakir Military Hospital, Department of Cardiovascular Surgery, Diyarbakir, Turkey

## Dear Sir

The publication by Alavi SM *et al.* highlights a subject with an ongoing debate, namely the ‘crsytalloid-colloid and colloid–colloid use following cardiac surgery’.^1^ They designed a randomised, double-blind clinical trial and compared the effects of three different solutions; 0.9% Ringer’s lactate, 4% gelatin and 6% hydroxyethyl starch (HES) solution. They concluded that the HES solution was better in terms of the volume-expanding effect; lower amounts were required compared to the other two solutions, and short-term renal functions were better.

We feel that there are several insufficiencies about the design and contents of the study. We believe that the process of double-blinding is quite challenging in this study, because the anesthesiologist or intensivist should be unaware of the solution administered. The process of double-blinding and how un-blinding was avoided should be detailed.

The haemodynamic status of the patients was defined using parameters such as cardiac index, which we think is very helpful, and also systolic and diastolic blood pressure levels of the patients. Unfortunately, no information regarding the use of inotropes intra-operatively and postoperatively was given. Indications for the use of inotropes were defined, but information on which type, in what dose and on how many patients they were used was not mentioned. This particular variable has great influence on the haemodynamic status of the patient as well the urinary output.^2^

Renal effects were analysed using serum creatinine and BUN levels, and urinary output. Besides the use of inotropes, the type of fluid used for cardiopulmonary bypass (CPB) priming, and the use of diuretics during CPB and in the postoperative period affect renal functions following cardiac surgery.^3^

Moreover, recent developments clearly demonstrated that the measurement of glomerular filtration rate (GFR) is the best overall index of renal function rather than measurements of creatinine and BUN levels alone. Serum creatinine levels do not increase until the GFR is reduced below 50%. 3

Finally, the reference by Boldt *et al.* was retracted in 2011.^4^ This reference should not be cited in this randomised trial, since Joachim Boldt, who published many articles on crystalloids and colloids, particularly in favour of HES solutions, was suspended for scientific misconduct.^5^

We believe that discussion on the use of HES solutions following cardiac surgery will continue, since there are many subjects on which consensus has not been reached. Randomised, double-blind clinical trials are the most valuable studies in the search for these answers but a good design and well-defined outcomes are required.
